# North Atlantic controls on wintertime warm extremes and aridification trends in the Middle East

**DOI:** 10.1038/s41598-017-12430-3

**Published:** 2017-09-26

**Authors:** Kondapalli Niranjan Kumar, Annalisa Molini, Taha B. M. J. Ouarda, Madhavan Nair Rajeevan

**Affiliations:** 1 Masdar Institute, Khalifa University of Science and Technology, P.O. Box 54224 Abu Dhabi, UAE; 2 INRS-ETE, Institut National de la Recherche Scientifique, Quebec, G1Y2T4 Canada; 3 Ministry of Earth Sciences, Prithvi Bhavan, Lodhi Road, New Delhi, Delhi, 110003 India; 4 Present Address: Atmosphere and Ocean Research Institute, University of Tokyo, 5-1-5, Kashiwanoha, Kashiwa-shi, Chiba 277-8564 Japan

## Abstract

The Middle East is one of the most water stressed regions in the world, receiving the majority of its hydrological input during the winter, in the form of highly variable and scattered precipitation. The persistence of wintertime anticyclonic conditions over the region can deflect storm tracks and result in extended spells of exceptionally hot weather, favoring prolonged droughts and posing a major threat to the already fragile hydrological equilibrium of the Middle East. Despite their potential impacts on water-security, winter warm spells (WWS’s) have received far less attention than their summer counterparts, and the climatic drivers leading to WWS’s onset are still largely unexplored. Here, we investigate their relationship with the internal modes of variability in the Atlantic Ocean, already known to influence winter circulation and extremes in Eurasia and Northern America. We show that the occurrence of WWS’s is strongly correlated with Atlantic variability over decadal time scales. To explain this correlation, we propose a teleconnection mechanism linking Atlantic variability to WWS’s via the propagation of Rossby waves from the North Atlantic pool, and the mediation of the Mediterranean circulation – thereby providing a basis to better predict future warming and aridification trends in the Middle East.

## Introduction

The Middle East is a predominantly water-scarce region that encompasses very diverse aridity levels and climatic conditions, ranging from the hot deserts of the Arabian Peninsula to the cool highlands of mountain ranges in Turkey and Iran. From an historical prospective, conflicts, diffuse political instability and, more recently, fast population growth have further aggravated the endemic water scarcity of this region^1^, thus exacerbating its vulnerability to climate change and climate variability in general^2,3^. Paradoxically the climate of the Middle East has been considered for a long time insensitive to anthropogenic climate change due to different masking effects associated with internal climate variability^4–6^ that are now better understood^7^. Only in recent years, climate projections and regional climate model simulations have revealed a consistent warming trend over the Middle East and the Eastern Mediterranean^8–10^, accompanied by a prevalent drying of the region^2,11,12^. Model results are widely supported by observational studies at the regional and sub-regional scales^13,14^, pointing in particular to the increasing frequency and intensity of hot extremes^15–17^, and underscoring the need to improve seasonal prediction skills over the region.

The core of these studies is centered on the summer months, due to the strong societal impacts that extreme temperatures are expected to produce during the hot season, especially when associated with elevated values of relative humidity and atmospheric pollution^18^. However, the majority of Middle Eastern countries already possesses an elevated degree of resilience to high temperatures, which during the summer can yet peak at as high as 45–50 °C in the Persian Gulf Region, Southern Iraq and Southwestern Iran^10,19^. Winter warm extremes, in contrast, have attracted a more limited attention in the literature, despite their potential impacts on the surface energy budget and the already precarious hydrological regime of the region^20^. Winter warm spells (WWS’s) affect the hydroclimatology of the Middle East both at synoptic and local scales, being strongly associated with quasi-stationary anticyclonic systems able to deflect Mediterranean cyclones tracks, and influence local hydroclimatic patterns alike.

In addition to this climatic forcing, the rapid soil desiccation that accompanies WWS’s exerts a limiting effect on evapotranspiration, potentially enhancing summer heat waves through intraseasonal feedbacks^21^.

Consequently, if summer heat waves better fit the classic definition of extreme event – producing sudden and intense societal impacts and higher absolute temperatures^9^ – WWS’s hold a more prominent role in regulating the exchange of energy and water at the interface between land and atmosphere. Also, they can modulate the hydrologic regime of the region, with a greater potential for shaping the longer-term aridification patterns and socio-political resilience of Middle Eastern countries^22–24^. From here the necessity to better understand the climate processes causing and sustaining WWS’s in arid and hyperarid regions like the Middle East.

We focus on the connection between the WWS’s and North Atlantic variability, one of the main drivers for climate in the Northern Hemisphere, whose role in the Middle Eastern hydroclimatology is, however, still poorly understood. Till now, in fact, the link between winter extremes in the Northern Hemisphere and North Atlantic variability has been mainly explored focusing on a different category of extreme events, i.e. the cold extremes in Europe and in the continental U.S.^25,26^. At the same time, some authors pointed out how major winter extremes like the 2009–2010 and 2010–2011 cold waves in Europe, Russia, and the U.S. coincided with the more extended and intense winter warm extremes affecting the Eastern Mediterranean, Middle East and Southwest Asia^20,26^. This connection between winter warm-extremes and prominent modes of natural climate variability is however largely unexplored, and classic predictors of cold weather in the Northern Hemisphere’s mid-latitudes such as the North Atlantic Oscillation (NAO) could have a more marginal role in the genesis of winter hot-weather^20^.

In this contribution, we show that the slow – interannual to decadal – scales of variability of WWS’s in the Middle East are strongly correlated with observed multidecadal modes of variability in the Atlantic. To explain this coupling, we propose a simple physical mechanism linking the WWS’s to the Atlantic variability through the subseasonal propagation of Rossby waves and the modulation of the Mediterranean circulation at sub-decadal scales. Given the still limited skills of climate models in reproducing the slow modes of climate variability, like the Atlantic multidecadal variability (AMV) and the relative contribution of internal and external forcing^27,28^, we base our analysis on analyzed fields from the NCEP-NCAR reanalysis over the period 1948–2016. Although a similar observational approach presents a number of limitations – and firstly the limited temporal span covered by the data set – it still represents the most robust approach to diagnose possible long term couplings between the AMV and the onset of WWS’s. Further details on the analyzed data-sets and diagnostic/statistical methods are provided in the *Materials and Methods* Section.

## Defining winter warm spells

Our analysis is based on a regional climatology of WWS’s over the Middle East from NCEP-NCAR reanalysis fields^29^. Reanalysis temperatures are tested against an analogous climatology extracted from the Global Historical Climatology Network (GHCN) land-based observations for the period of maximum station coverage over the study region (1979–2016)^30^. The goal of this preliminary intercomparison is to assess whether statistics inferred from the reanalysis fields, including extreme value statistics, are consistent with the ones obtained from station data to further extend our analysis throughout the entire NCEP-NCAR reanalysis operational period (1948–2016). Station time series were selected among the ones with maximum temporal coverage (at least 75%) over the entire observational period (1979–2016).

**Figure 1:**
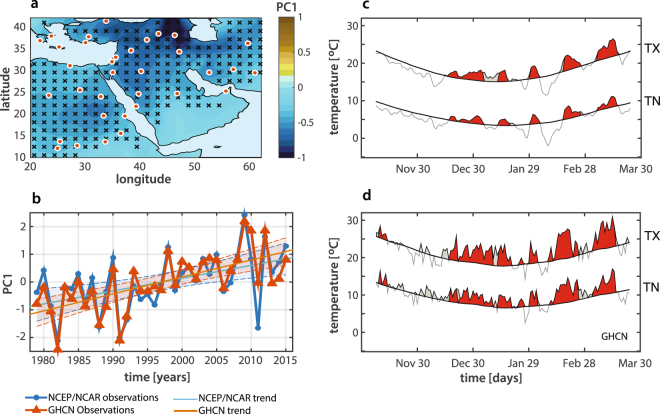
Winter temperature climatology and WWS’s in the Middle East as defined from NCEP/NCAR and GHCN data. (**a**–**b**) Spatial patterns and temporal trends of average winter temperatures over the Middle East: (**a**) First principal component (PC1) of the mean winter (NDJFM) temperature (1948–2016) from NCEP/NCAR reanalysis (contours) and spatial distribution of meteorological stations (GHCN, orange/red dots). (**b**) Normalized PC1 from reanalysis (blue) and meteo-stations (red) for 1979–2016 winters with the corresponding trends and confidence bands at the 95% confidence level. (**c**–**d**) Winter 2009–2010 warm spells as obtained from reanalysis (**c**) and station (**d**) data. Thick black lines indicate the mean daily 95^th^ percentile of TX and TN over the study area; warm spells are indicated by red shading.

The study region – comprising North-eastern Africa, the Eastern Mediterranean, the Arabian Peninsula and Persia – is identified based on the first two principal components of NCEP-NCAR and GHCN temperatures (Fig. 1a, black crosses) as the homogeneous area for which the first two principal components of winter temperature explain at least 60% of the total variance (see *Materials and Methods*). The spatial variability of winter temperatures over the Middle East is yet well represented by the the first principal component (PC1) of NCEP/NCAR temperature fields (Fig. 1a, shaded contours), and displays a sharp North-South gradient over the Arabian Peninsula, highly consistent with previous studies reporting a similar winter North to South positive gradient for both maximum (TX) and minimum (TN) temperatures^3^. Also, both the NCEP/NCAR and GHCN temporal PC1s obtained by projecting the eigenvectors into the spatially weighted anomalies of the study region (Fig. 1b) display a sharply increasing linear trend, significant at the 99% confidence level and an overall regional trend of 2.14 °C for GHCN observations, and 1.05 °C for NCEP/NCAR data. These trends in the winter temperatures are in agreement with previous observational studies^14^, also reporting a tendency to warming in daily TX and TN over the period 1950–2003, and with regional coupled model projections and ensemble results from CMIP5 climate models^9^ predicting a significant increase (2.0 °C to 3.1 °C) of mean winter temperatures over the Middle East by middle 21^th^ century.

In order to focus on winter warm extremes, we identify the WWS’s across the two data-sets based on a simple over-threshold quantile criterion, the duration, and the spatial extension of the extremes. Days for which both TX and TN – averaged over the homogeneous study region in Fig. 1a – fall within the upper 5%-percentile of the local climatology for the base period (1948–2016 for NCEP/NCAR and 1979–2016 for GHCN) are labeled as winter (NDJFM) warm extremes, and become part of a WWS (see *Materials and Methods*). Figure 1c and d portray the regional WWS’s (red area) for winter 2009/10, as extracted from NCEP/NCAR reanalysis and GHCN data respectively. Winter 2009/10 is considered one of the hottest and driest winters on record for the Middle East^31^, and five distinct hot-spells of different duration can clearly be identified in both the reanalysis and land-based observations. Over the Middle East, the brevity of observations and their sparse character make a comprehensive validation of reanalysis data with observations difficult^32^. However, there are strong qualitative similarities between observations and reanalysis fields in terms of main features, intensity and frequency of the hot spells, pointing to the fact that the main characteristics of WWS’s are realistically reproduced in the reanalysis. These consistent results across the two data-sets encourage us to extend our analysis to the entire operational period of the NCEP/NCAR reanalysis (1948–2016).

## Results

### Interannual and decadal variability of WWS’s

We first explore the temporal evolution of WWS’s over interannual to decadal scales, and their possible connection with the internal variability modes of the Atlantic Ocean. Interannual temperature variability over the Middle East has been previously associated with the states of the North Atlantic Oscillation^5,33^. However, NAO is considered a weak predictor of the Middle Eastern temperature regime over longer, decadal scales^5,20^. The long-term variability of the WWS’s is here quantified in terms of density of occurrence of the extreme temperatures (number of occurrences per season) and duration (total number of days above threshold per season; as detailed in the *Materials and Methods* Section).

**Figure 2:**
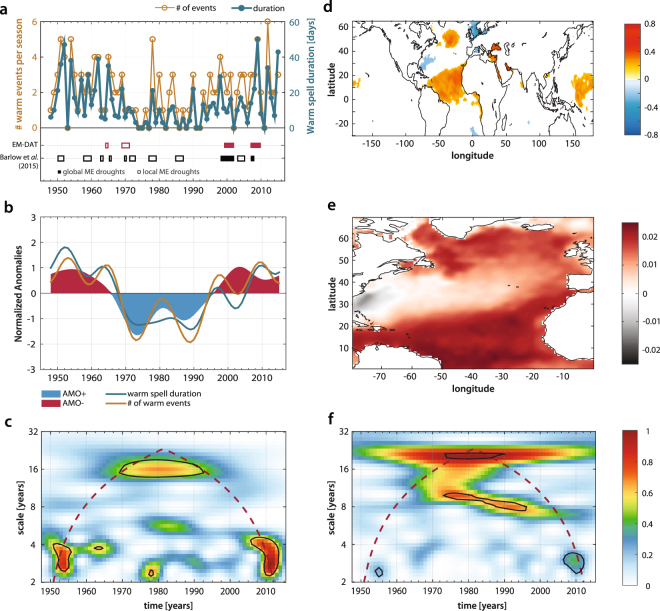
Co-evolution of Atlantic SSTs and Middle Eastern WWS’s over interannual to decadal scales. (**a**) Number and duration (days) of the WWS’s over the period 1948–2016 (top) and occurrence of major historical droughts in the region (local and global) from EM-DAT database^34^ and Barlow *et al*.^31^ (bottom). (**b**) Comparison between the temporal evolution of the WWS’s (occurrence and duration) over the study period and winter (NDJFM) Atlantic Multidecadal Variability (AMV)^36,37^ after normalization and rescaling. (**c**) Wavelet power spectrum of the WWS’s frequency of occurrence in (**a**) after detrending. Black contours identify pointwise significant wavelet coefficients at the 95% confidence level^39^ [See *Materials and Methods*]. (**d**) Spatial correlation of the occurrence of WWS’s with global SSTs. Panel (**d**) only shows values significant at the 95% confidence level. (**e**) Spatial patterns of the North Atlantic SST leading mode obtained from principal component analysis. (**f**) Morlet wavelet spectrum of North Atlantic PC1 time series after detrending. Significance test as in (**c**).

Between winter 1948 (ND48–JFM49) and winter 2015 (ND15–JFM16), both frequency and duration of the WWS’s (Fig. 2a) show a strong interannual variability with a sharp decreasing trend between the 50’s and the 70’s, an intermittent low-phase between the 70’s and the 90’s, and a significant increasing trend after 1990. This decadal variability pattern seems to support the existence of a prevailing masking effect of natural climate modes on anthropogenic climate change over the region, before the early 90’s^5^. In contrast, the increasing trend after 1990 corresponds to a period of climate change amplification, manifesting itself both in terms of extremes and average temperature regime in the Middle East^7^ (see Fig. 1b).

The increasing trend in both the frequency and the duration of WWS’s through the last three decades is further reflected in the co-occurrence of two important mega-droughts (1998–2002 and 2007–2009 circa) impacting the entire study region^31^ (Fig. 2a, bottom), in that both local and regional droughts seem to be preceded by anomalously hot and dry winters. Also keeping in mind that data on the frequency and duration of intense droughts in the Middle East are sparse^31,34^, the apparent increasing intensity, duration and spatial extent of Middle Eastern droughts in the last decades is documented in a number of studies^23,24,31^, and the period 1998–2012 has been recently described as the most intense mega-drought of the last 900 years in the Levant, based on the Old World Drought Atlas (OWDA) tree ring reconstruction^35^.

Also, the decadal trends we observe in both the occurrence and duration of WWS’s appear to be significantly correlated with multidecadal variability in the Atlantic Ocean (AMV, also known as Atlantic Multidecadal Oscillation, AMO^36^), displaying a strong positive phase between the 30 s and the 60 s, followed by a negative phase (1965–1985 circa) and a new positive phase after 1990 (Fig. 2b). The winter AMV index is computed here by averaging monthly sea surface temperature (SST) anomalies over the North Atlantic [75–7 W; 25–60 N] from December to March, and detrended by subtracting global SST anomalies^37^. Also, all three time series shown in Fig. 2b (AMV, WWS occurrence and duration) are smoothed for visualization purposes through a Lanczos low-pass filter to remove high frequency variability, and rescaled through a simple normalization procedure (see *Materials and Methods*).

The signature of the slow modes of variability of the North Atlantic Ocean is also evident in the wavelet power-spectrum of the unsmoothed detrended WWS occurrences (Fig. 2c). Spectral power peaks falling within the 95% confidence region (black contour) are identified through a classic point-wise significance test against a red background noise based on statistical bootstrap^38,39^. Here we can detect two main spectral signatures: one decadal (significant spectral power at scales greater than 10 years), weaker but yet significant, and one subdecadal (localized within the 2–7 years band), displaying a stronger characteristic signature. The relative role of of these two spectral components is discussed in the following sections in the contest of the decadal and subdecadal dynamics of WWS’s.

### Atlantic modulation of the winter circulation over the Middle East

**Figure 3:**
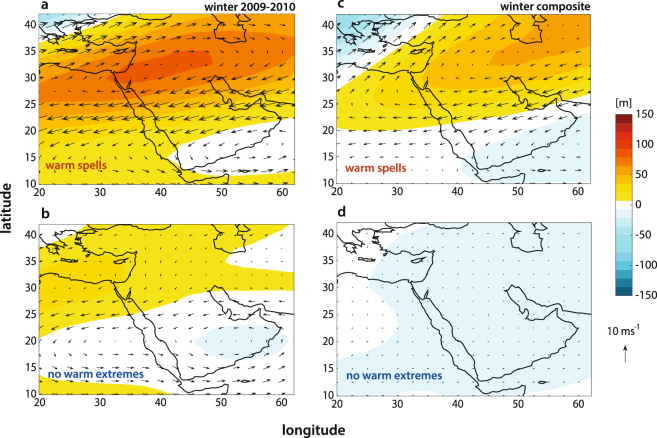
Mid-troposphere anomalies of geopotential heights associated with WWS’s as compared to average winter conditions. (**a**–**b**) 2009–2010 winter composites of daily 500 hPa geopotential height anomalies during warm spells (**a**) and average winter conditions (**b**). (**c**–**d**) Same as (**a**–**b**) but for the entire study period (1948–2016 composite).

WWS’s are associated with extended winter blocking systems that can both alter the dynamics of regional storm tracks by controlling the propagation of stationary waves^40^, and sustain the enhancement of land-atmosphere interactions such as the soil moisture-temperature coupling^41,42^. The mechanisms through which persistent anticyclonic patterns can favor the genesis of the WWS’s is reveled by the composites of mid-troposphere (500 hPa) geopotential height anomalies during the WWS’s as contrasted with synoptic conditions in absence of WWS’s (Fig. 3). Figure 3a–b, show the geopotential composites for the 2009–2010 WWSs (a) versus the composite of average winter weather days, while panels c–d report the composite for the entire study period (1948–2016) for hot spells (c) and average winter conditions (d). In both cases, sharp anticyclonic patterns centered on the Northern part of the Arabian Peninsula characterizes the days associated with the WWS’s, while non-extreme days show the absence of any synoptic characteristic signature.

To provide further insight into the co-evolution of North Atlantic SSTs and WWS’s in the Middle East, it is therefore crucial to investigate the possible connections between SST anomalies and the development of the extended and persistent anticyclonic patters over the region. Coherent, large-scale SST anomalies are known to be closely related with quasi-stationary anticyclonic patterns and atmospheric blocking^43,44^, and with heat waves and warm extremes in general. A prominent example of this connection is provided by the Central Europe mega-heat wave of summer 2003, which a number of authors linked to the dynamics of SSTs in the Indian Ocean and the Mediterranean^45^, and to the effect of anomalous diabatic heating in the tropical Atlantic^44^. Similarly, the Russian heat wave of 2010 has been associated with the evolution of SSTs in the Northern Indian Ocean^46^ and with internal atmospheric variability^47^.

To investigate the connection between WWS’s and SST anomalies we analyze the correlation between the occurrence of the winter extremes over the Middle East and global SST data from HadISST^48^ after detrending and averaging over the winter season.

The resulting correlation map (Fig. 2d) shows the areas where correlation values are statistically significant at the 90% confidence level based on a Student *t*-test. SST anomalies over the North Atlantic and the Mediterranean show a strong correlation with the winter warm spells. Additionally, Fig. 2d reveals the typical tripole structure over the Atlantic with significant correlation in the subpolar region and in the tropical Atlantic Ocean and negative correlation in between. This tripolar SST pattern predominantly emerges during the boreal winter, and is characterized by three action centers of alternative polarity in the subpolar-tropical Atlantic^49–51^. The SST tripole is belived to respond to atmospheric forcing associated with the NAO^50^, although the tripole itself can induce a NAO-like atmospheric response^52^. However, the winter SST tripole is essentially a leading mode of low frequency SST variability, as can be easily shown by decomposing the signal into its dominant modes. At this goal, we applied empirical orthogonal function (EOF) decomposition to the region encompassing 0–70 N and 80–0 W (partially overlapping the AMV region, but without detrending with respect to global average temperatures^37^) for the period 1948–2016. The spatial pattern of the EOF1 is shown in Fig. 2e. It displays a clear tripole structure similar to the one discussed above and explains nearly 34% of the total variance. Also, the wavelet power spectrum of the detrended EOF1 time series averaged over the North Atlantic (0–70 N and 80–0 W, Fig. 2f) displays a significant power in two distinct bands centered at 18 years and 8 years respectively. The 18 years band is also present in the WWS’s occurrence power spectrum (Fig. 2c) and is determined by the alternation between positive (50’s to 60’s and starting in the 90’s) and negative (between the 70’s and the 90’s) oscillations characterizing both the tripole mode and WWS’s. Additionally the WWS’s in the Middle East are also significantly linked with anomalous SSTs in the Mediterranean and the Black Sea, giving rise to a further bipolar structure over the Mediterranean basin (Fig. 2d).

### The role of the Mediterranean Sea

The coupling between the WWS’s and the decadal variability in the North Atlantic could either originate from a large scale atmospheric forcing or more local sea-atmosphere interaction processes. The tripolar correlation structure in the Atlantic, and the bipolar structure in the Mediterranean (Fig. 2d) seem indeed to indicate a joint action of synoptic (originating in the Atlantic) and more regional (from the Mediterranean) sea surface-atmosphere interaction mechanisms. North Atlantic conditions are known to exert a strong influence on the evolution of Mediterranean SSTs^53^. In this context Mediterranean SST spatiotemporal variability has been previously connected to the increased temperature of the Atlantic inflow^54^, while Mediterranean SST multidecadal variations are known to be highly correlated with the state of the North Atlantic indices^54–56^. In particular, it has been hypothesized that multidecadal variability in the Atlantic Ocean is transmitted to the Mediterranean Sea via atmospheric processes^56^.

**Figure 4:**
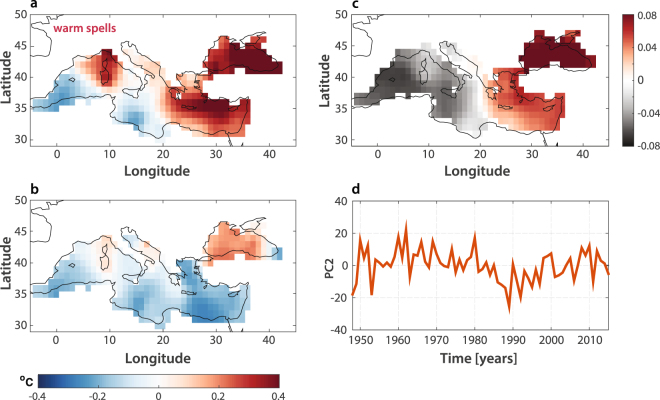
Mediterranean SSTs modulation of WWS’s. (**a**) Composite winter SSTs during the warm episodes of 1950–1965, 1975–1980, and 2005–2013, corresponding to the significant spectral power peaks observed in the 2–7 years period band of Fig. 2c. (**b**) Same as (**a**) but for winter conditions not forced within the 2–7 years band. (**c**–**d**) Spatial patterns and time evolution of the PC2 of winter Mediterranean SSTs during the period 1948–2016.

A similar mechanism could also explain the synchronization between the slow-modes of North Atlantic variability and the occurrence of WWS’s, through the mediation of Mediterranean SSTs excited at both decadal and subdecadal scales by the AMV. At multidecadal scales (>40 years) Atlantic and Mediterranean SSTs are considered highly coherent^56^. Additionally, their co-spectrum has been found to display significant secondary coherence peaks at around period ~18 years and in the 2–7 years spectral band^56^. The significant spectral power band centered around 18 years seems also a robust feature of both AMV and WWS’s spectral signatures (Fig. 2c,f). We thus postulate that this slow mode of variability could be passed across from the North Atlantic to the Middle East through the filter of the Mediterranean basin. While the AMV does not show any significant spectral peak at subdecadal scales other than ~8 years, the North Atlantic Oscillation seems capable of exciting the anomalous Mediterranean SST modes within the 2–7 years spectral band^56^. Therefore, the subdecadal spectral peaks at 2–7 years characterizing the occurrence of WWS’s (Fig. 2c) may be linked to higher-frequency responses of Mediterranean SSTs to the Atlantic forcing. In support of this thesis, the composite Mediterranean SST anomalies for the years displaying a significant spectral signature in the 2–7 years band (1950–1965, 1975–1980, and 2005–2013, Fig. 4a) show a clear bipolar structure (similar to the correlation pattern in Fig. 2d) with a cooler Western Mediterranean and an exceptionally warm Eastern Mediterranean. This bipolar pattern, in contrast, is absent from the composite of the years not forced in the 2–7 years band (Fig. 4b). Therefore, the observed warming of Eastern Mediterranean (Fig. 4a) seems to have a strong link with the subdecadal dynamics of WWS’s over the Middle East.

Elevated land surface temperatures in combination with the warming of the surface of the Eastern Mediterranean sea would be expected to increase latent and sensible heat fluxes. At the same time, the increased stability of the atmospheric conditions associated with the occurrence of WWS’s (Fig. 3) tends to restrict vertical heat fluxes to the lower part of the boundary layer, further trapping warm air masses within the lowermost atmosphere due to reduced mixing^42^. The EOF1 component of the Mediterranen SSTs is not subject to any seasonal effect and it mainly responds to the forcing at interdecadal scales^57^.

In contrast, the interannual variability of the Mediterranean SSTs is mainly explained by their EOF2, and previous studies have highlighted how the spatial EOF2 is characterized by a zonally oriented dipole with oppositely directed changes in the western and eastern Mediterranean Sea^57^. The spatial EOF2 of the winter Mediterranean SSTs over the study period (1948–2016) also shows a marked bipolar structure (Fig. 4c), while the temporal evolution of the EOF2 averaged over the Mediterranean basin (Fig. 4d) appears to be strongly variable at interannual scales (comparable with the variability within the 2–7 years period band). Consequently, warmer SSTs over the Eastern Mediterranean, enhanced evaporation, and the strong anticyclonic structures prevailing over the Eastern Mediterranean and Middle East, all contribute to suppress deep convection and trigger and sustain the WWS’s. The Mediterranean EOF2 hence appears to be a robust predictor of WWS’s at interannual scales.

### Linking subseasonal planetary waves to Middle Eastern WWS’s

**Figure 5:**
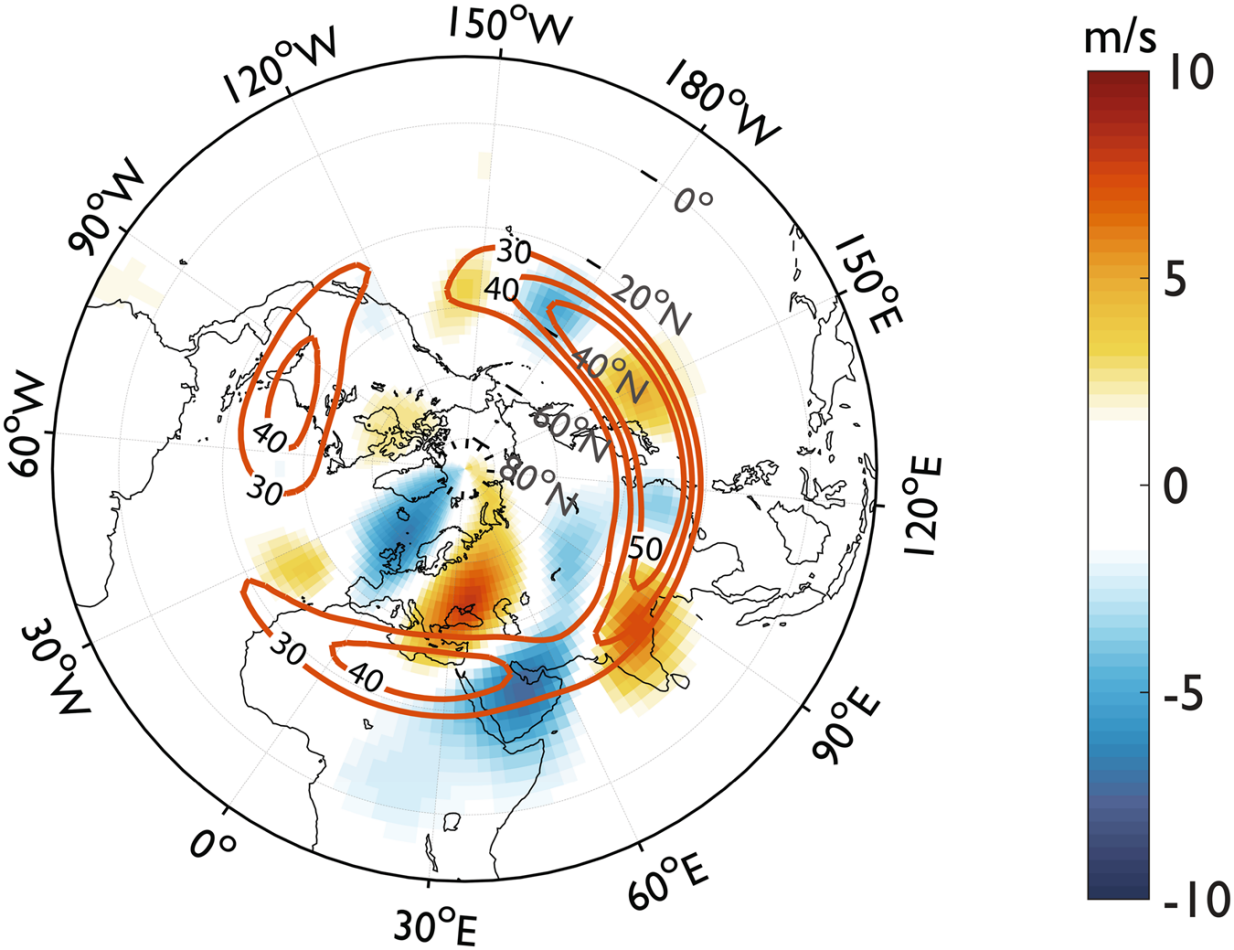
Rossby waves developing in the North Atlantic during winter warm-spells as they merge into the Middle Eastern jet stream. Composite meridional wind (m/s, filled contour) and zonal winds (m/s, orange contour) anomaly at the 200 hPa pressure level for the 1948–2016 winter warm events over the Middle East.

We have seen that Atlantic and Mediterranean SSTs could play a major role in controlling WWS’s over the Middle East. Large and persistent Atlantic SST anomalies seem in fact to modulate the occurrence of the WWS’s at interannual and decadal scales through the mediation of the Mediterranean SSTs, creating the conditions for the development of extended and persistent anticyclonic structures over the region. In turn, anticyclonic anomalies observed during warm extremes are often part of global-scale stationary Rossby wave trains^44,58–60^, so that the physical explanation of the teleconnection between modes of variability in the Atlantic and Middle Eastern climate during the evolution of warm spells could be found in the propagation of planetary wave trains from the North Atlantic pool down to the latitudes of the Middle Eastern jet stream. To better explore this thesis, we analyze the meridional wind anomalies at 200hPa (v200hPa) across the study period, considered good indicators of the Rossby wave activity. Previous studies linked the equatorial Pacific SSTs with the Middle Eastern winter climate through the Rossby wave propagation^61–63^. We construct here the composite v200hPa anomalies for all the WWS’s (131 events, corresponding to 840 warm days) during the study period, and we overlap it with the zonal wind composite at 200hPa (u200hPa, Fig. 5). A marked Rossby wave train pattern originating in the North Atlantic Ocean and encountering the Middle East jet stream in the subtropics, is clearly emerging from the composite of the WWS’s in Fig. 5. Here, planetary waves show a wide amplitude (large meridional component) and a dominant wave number of 6 across the diverse warm events (see supplementary Fig. S1), similarly to the quasi-resonant Rossby waves that have been associated with extreme events in the Northern Hemisphere^60^. The teleconnection between the North Atlantic modes of variability and the winter warm extremes in the Middle East could hence be explained by the Atlantic forcing on Rossby waves, leading to the onset of a wave train of disturbances and resulting in a strong teleconnection downstream of the local heat source. It is also interesting to note that, during the winter, the subtropical jet stream can act as a wave-guide for the planetary waves in the Northern Hemisphere^64^. Through this wave-guide, Rossby waves can further propagate downstream to East Asia and the North Pacific Ocean along the subtropical jet stream.

## Discussion and Conclusions

Our results suggest that both Rossby wave subseasonal patterns and the high frequency variability in the Mediterranean SSTs can provide a strong basis for the seasonal prediction of WWS’s in the Middle East, helping to better understand future warming and aridification trends in the region. Our findings are based on the Middle Eastern winter temperature climatology extracted from the NCEP-NCAR reanalysis fields and covering the period 1948–2016. Whether the span of the available observations/reanalysis fields^32^ could limits our long-term prognostic skills, the dependence of WWS’s from the slow modes of climate variability in the Atlantic Ocean appears as a robust feature of the Middle East climatology, and poses the bases for an improved seasonal prediction of winter warm extremes. A further expansion of this analysis could explore the future long-term dynamics of WWS’s in climate model projections, although with a caveat. Climate model skills in reproducing slow modes of climate variability like AMV are in fact still limited, and a number of concerns have been recently raised for example, on the capability of the member of the CMP5 ensemble to simulate the relative magnitude of internal variability and external forcing components, and their coupling^27,28,65^. For this reason, reanalysis and observations, although limited in prognostic scope, can be currently considered one of the most robust tools to investigate the link between winter warm spells in the Middle East and multidecadal variability in the Atlantic Ocean.

## Materials and Methods

### Observations

#### Temperatures in the Middle East

Data used in this analysis consist of daily maximum and minimum temperatures (TX and TN) extracted from the NCEP-NCAR reanalysis surface air temperature fields^29^ for the months of November to March (extended winter season). They span the period 1948–2016 and are available on a Gaussian T62 grid. The latitudinal grid spacing varies while preserving equal areas and is approximately equal to 1.9°, while longitudinal spacing is 1.875°. In order to evaluate how WWS’s are reproduced in the reanalysis, we also obtained TX and TN daily observations from the National Climatic Data Center (NCDC) Global Historical Climatology Network^30^ (GHCN). The GHCN dataset contains TX and TN for nearly 80,000 stations around the globe^30^, from which we selected the subset of stations located in the Middle East (see Fig. 1a) whose records fall within the period 1979–2016. The 1979–2016 time span corresponds in fact to the highest network density over the Middle East. GHCN air temperature data have been extensively quality controlled^66^ and have been analyzed in a variety of different studies^67^.

#### Synoptic conditions during the WWS’s

Composite synoptic conditions (mean conditions and anomalies) characterizing the onset and evolution of WWS’s over the Middle East are quantified based on NCEP/NCAR reanalysis data^29^. For this purpose, we obtained the daily zonal and meridional wind fields, vertical velocity, and geopotential height, at standard pressure levels available with a 2.5° × 2.5° horizontal resolution, for the period 1948–2016. The daily anomalies were calculated by subtracting the climatological daily mean for the reference period 1981–2010 during the winter season.

#### Sea surface temperatures and dominant modes of variability in the Atlantic Ocean

Persistent sea-surface temperature (SST) anomalies are known to be linked to large-scale circulation patterns, like the ones leading to synoptically extended geopotential anomalies, although the exact connection between SST anomalies and warm extremes is not completely understood – especially in the Middle East. The role of Atlantic and Mediterranean SSTs in controlling extreme heat conditions during the Middle Eastern winter is here assessed based on the Hadley Centre Sea Ice and SST dataset (HadISST)^48^. Monthly 1° × 1° resolution global SST data from 1870 onward are available from the HadISST repository. The dataset is constructed using a reduced spatial optimal interpolation procedure from the measured SST values compiled from the International Comprehensive Ocean Atmosphere Data Set (ICOADS) database and the Met Office Marine Data Bank. Here, sea ice data are obtained from a variety of sources including digitized sea ice charts and passive microwave retrievals. Also wintertime Atlantic multidecadal variability (AMV) is constructed using the HadISST dataset over the period 1948–2016. The monthly SST anomalies are determined with respect to the 1981–2010 climatology, then the winter AMV index is computed by averaging the monthly SST anomalies over the North Atlantic [75–7 W; 25–60 N] from December to March (DJFM)^68^. SST global anomalies are subtracted from the index to remove the global warming trend and the influence of tropical oceans, as previously suggested by other authors^37,69^. A Lanczos low-pass filter with 21 total weights and a threshold of 10 years is applied to the AMV and to the WWS occurrence and duration time series plotted in Fig. 2b to remove high-frequency variability. End points of each time series are reflected to avoid data losses^69^.

### Identification of the WWS’s

Absent an accepted standard for defining heat extremes/heat waves^70^, we adopt here a simple procedure based on the spatial and temporal variability of temperature to identify WWS’s, as well as their duration and frequency of occurrence. WWS’s are identified based on the local winter (NDJFM) climatology over the base period 1948–2016. Both maximum (TX) and minimum (TN) temperatures are used in our assessment (i.e. temporal and spatial constraints need to be consistently met for both TXs and TNs). The same procedure is applied to reanalysis data (NCEP/NCAR) and observational data (GHCN).

#### Regional winter heat spells are then defined based on the following three steps

(a) Spatial extension: The WWS’s are defined as regional extremes, and over-threshold values are estimated based on the daily TXs and TNs averaged over the selected study region. Analogously, the local 95^th^ percentile thresholds (TX95 and TN95) used to extract over-threshold values (see point (b) in this section) are defined at the regional scale (entire study area). The study area which includes North-eastern Africa, the Eastern Mediterranean, the Arabian Peninsula and Persia, is selected by applying principal component analysis (PCA) to winter (NDJFM) mean temperatures comprising the region 10°–45°N and 20°–65°E over the period 1948–2016. Pixels/station points for which the first two PCs explain more than 60% of the total variance are included in the study area (identified by black asterisks in Fig. 1a).

(b) Temperature threshold: A hot day is defined as the day where daily regional TX and TN values exceed the climatological (1948–2016) daily upper 95^th^ percentile for both maximum and minimum temperatures. The daily regional 95^th^ percentile is computed for each day D using temperature data over the entire climatology (68 years) between D -7 days and D +7 days. For example, to compute the 95^th^ percentile for December 8^th^, we use the temperature climatology between December 1^st^ and 15^th^. This method follows closely the approach adopted in a number of studies on warm extremes and heat waves identification^71,72^.

(c) Temporal extension: The minimum temporal extension of the regional WWS’s over the Middle East is of 3 consecutive days, during which both TX and TN must exceed daily TX95 and TN95.

### Patterns identification and spectral methods

The spatial and temporal modes of variability characterizing different climatic modes and the winter temperature regime of the Middle East are identified through the use of the Empirical Orthogonal Function (EOF) decomposition (a.k.a. Principal Components Analysis, PCA). Spatial EOFs are obtained by computing the eigenvalues and eigenvectors of the covariance matrix of the different field variables^73^. Analogously, the time series of each mode are estimated by projecting the derived eigenvectors onto the anomalies of winter temperatures spatially weighted over the study region^73,74^.

Wavelet power spectra are obtained from detrended time series through the continuous convolution of the analyzed signal with a Morlet wavelet basis function with central frequency *ω*_0_ = 6. Areas of significant spectral power (at the 95% confidence level) are identified through a classic point-wise significance test against a red background noise based on statistical bootstrap^38,39^.

### Data availability

All the data used in this analysis are publicly available. NCEP-NCAR reanalysis fields can be obtained from the NOAA repository at http://www.esrl.noaa.gov/psd/data/gridded/data.ncep.reanalysis2.html. Daily temperature data from the Global Historical Climatology Network-Daily database are available from http://www1.ncdc.noaa.gov/pub/data/ghcn/daily. Monthly values for the Atlantic Multidecadal Variability index between 25–60 N and 75–7 W can be obtained from the KNMI data explorer at http://climexp.knmi.nl. HadSST3 (version 3.1.1.0) SSTs are available from Metoffice at http://www.metoffice.gov.uk/hadobs/hadsst3/data/download.html.

## Electronic supplementary material

Supplementary Information Figure S1
