# Supplementary material for: North Atlantic controls on wintertime warm extremes and aridification trends in the Middle East
Source: Sci Rep. 2017 Sep 26;7:12301. doi: 10.1038/s41598-017-12430-3 (PMC5615055; doi:10.1038/s41598-017-12430-3)
Supplement: Supplementary file 1 — Supplementary Information Figure S1 [file 41598_2017_12430_MOESM1_ESM.pdf]

North Atlantic controls on wintertime warm extremes and aridification trends in the Middle East

Kondapalli Niranjan Kumar[1,2,*], Annalisa Molini[1,*], Taha B.M.J. Ouarda[1,3], and Madhavan Nair Rajeevan[4]

[1]Masdar Institute, Khalifa University of Science and Technology, P.O. Box 54224, Abu Dhabi, UAE
[2]Now at: Atmosphere and Ocean Research Institute, University of Tokyo, 5-1-5, Kashiwanoha, Kashiwa-shi, Chiba 277-8564 Japan
[3]INRS-ETE, Institut National de la Recherche Scientifique, Quebec, G1Y2T4, Canada
[4]Ministry of Earth Sciences, Prithvi Bhavan, Lodhi Road, New Delhi, Delhi 110003, India
[*]Correspondence to: niranjan@aori.u-tokyo.ac.jp, amolini@masdar.ac.ae

Supplementary Information

Supplementary Figure S1.

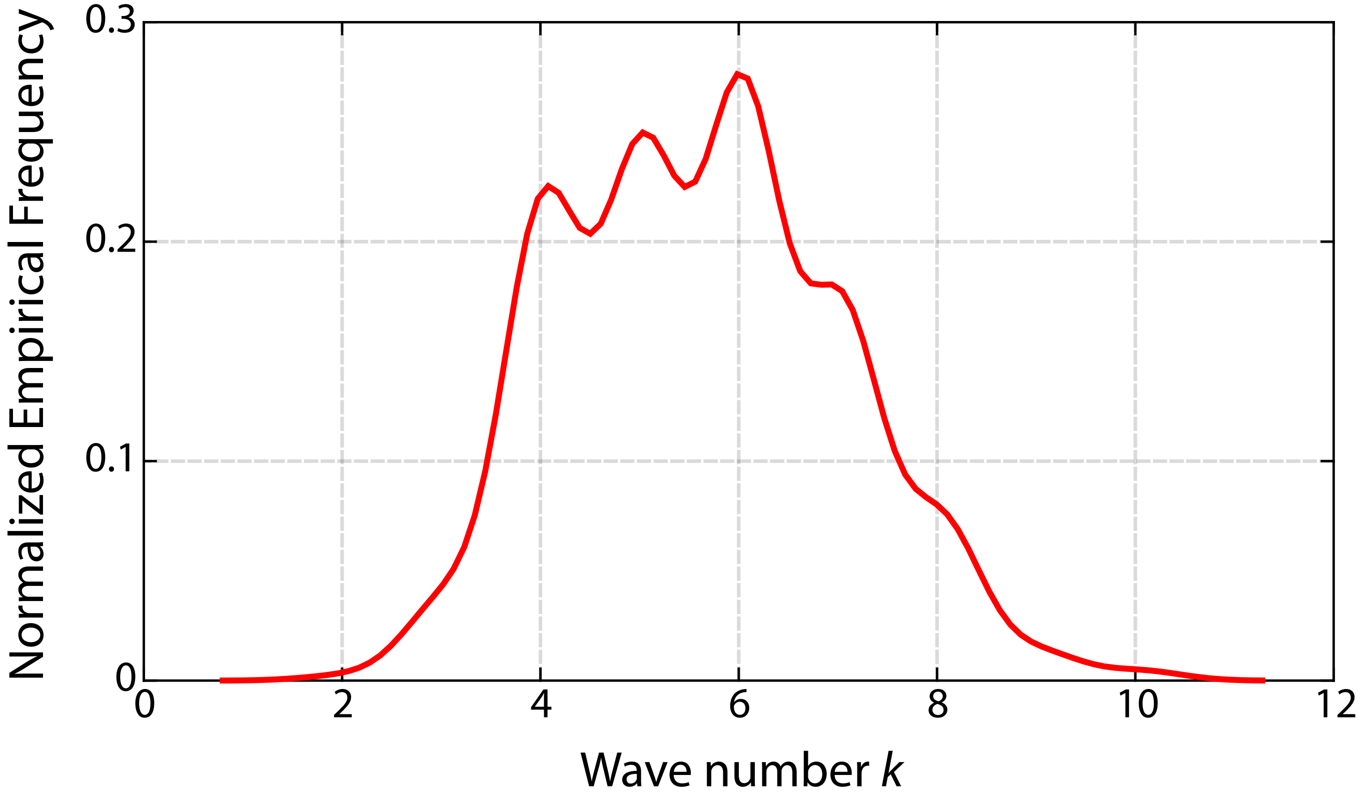

Figure S1. Dominant wave number for Rossby wave trains during WWS's.
